# Acute Encephalopathy Associated with Human Adenovirus Type 14 Infection in 7-Year-Old Girl, Japan

**DOI:** 10.3201/eid3102.241168

**Published:** 2025-02

**Authors:** Shinsuke Mizuno, Yoshihiko Tanimoto, Ai Mori, Tomoaki Fuseya, Yusuke Ishida, Masahiro Nishiyama, Azusa Maruyama, Masashi Kasai

**Affiliations:** Hyogo Prefectural Kobe Children’s Hospital, Kobe, Japan (S. Mizuno, Y. Ishida, M. Nishiyama, A. Maruyama, M. Kasai); Kobe Institute of Health, Kobe (Y. Tanimoto, A. Mori, T. Fuseya)

**Keywords:** adenovirus, acute encephalopathy, viruses, sentinel surveillance, Japan

## Abstract

Only 2 cases of human adenovirus type 14 (HAdV-14) have been reported in Japan since 1980. We report a 7-year-old girl with acute encephalopathy associated with HAdV-14 infection genetically similar to strains from the United States. The patient had not had contact with international travelers. HAdV-14 surveillance should be strengthened in Japan.

Human adenoviruses (HAdVs) are nonenveloped double-stranded DNA viruses that are relatively physicochemically stable (*1*). To date, >111 HAdV types have been recognized. Their clinical manifestations differ according to type and can result in respiratory, ocular, gastrointestinal, and urogenital diseases (*1*). Respiratory infections are often caused by HAdV types 3, 7, and 55 in East Asia, and most resolve spontaneously (*2*). Pneumonia caused by HAdV types 7, 8, and 55 is more severe (*2*,*3*). HAdV-14 was first identified as an acute respiratory pathogen in the Netherlands in 1955 but is rarely reported (*4*), although outbreaks have been reported in the United States and Europe (*5*–*7*). In Japan, the National Epidemiologic Surveillance of Infectious Diseases system conducts sentinel surveillance nationwide, but only 2 cases of HAdV-14 have been reported since 1980 (*8*). We report the case of a 7-year-old girl with influenza-like symptoms and acute encephalopathy associated with HAdV-14 infection that was genetically identical to strains isolated in the United States in 2019, despite the patient having no contact with international travelers.

A 7-year-old girl sought care at the Hyogo Prefectural Kobe Children’s Hospital (Kobe, Japan) for fever, cough, and seizures. She had a history of febrile seizures. No abnormalities were detected on electroencephalography. After her arrival at the emergency department, the generalized seizures continued and stopped ≈2 hours after onset after administration of multiple anticonvulsants. The patient was febrile (39.5 °C) with a pulse rate of 160–170 beats/min, respiratory rate of 28 breaths/min, oxygen saturation of 94% breathing room air, and a pediatric Glasgow coma scale of 9 (E4V1M4). The physical examination was otherwise unremarkable. Routine laboratory tests and a cerebrospinal fluid analysis revealed no abnormalities. Chest radiography revealed no evidence of pneumonia. Computed tomography of the head plane revealed no abnormalities, and electroencephalography showed slow wave activity. Multiplex PCR testing of a nasopharyngeal swab specimen was positive for HAdV. We diagnosed acute encephalopathy because the patient remained unconscious 6 hours after seizure onset. We initiated targeted temperature management for 48 hours, along with coma therapy using barbiturates. On day 9, magnetic resonance imaging of the brain revealed high intensity in the subcortical white matter and caudate nuclei bilaterally (Figure 1). On day 11, the patient was discharged with upper limb weakness. Outpatient follow-up at 1 month after diagnosis revealed no neurologic problems.

**Figure 1:**
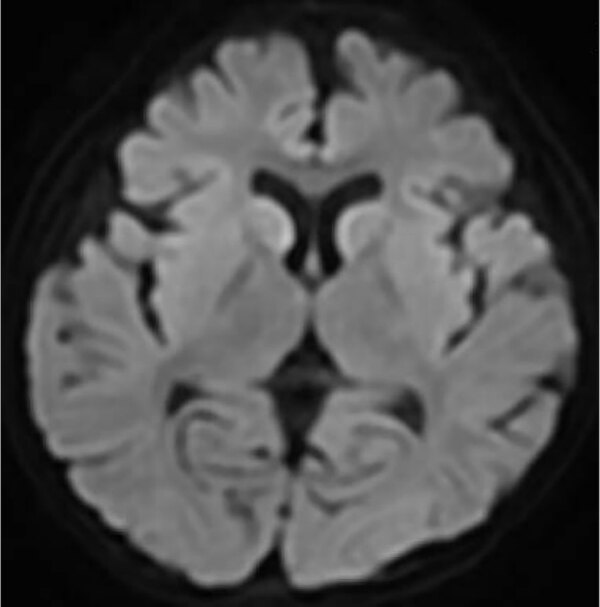
Diffusion weighted magnetic resonance imaging scan of brain in study of acute encephalopathy associated with human adenovirus type 14 infection in 7-year-old girl, Japan. Image shows high intensity in the subcortical white matter and bilateral caudate nuclei.

Viral cultures of nasopharyngeal swab and stool samples in A549 cells were positive for HAdV. Whole-genome sequencing showed that the isolated strain was the same clade as the first reported strain, de Wit strain, and highly similar to the strains reported during 2003–2019, differing from the most recent reported strains (identified in the United States in 2019) by only a single nucleotide variant (noncoding region) and a 2-nucleotide insertion (open reading frame 3 protein) (Figure 2). Results of PCR testing and viral culture of cerebrospinal fluid and blood samples were negative. Further questioning of the patient’s parents revealed no contact with international travelers.

**Figure 2:**
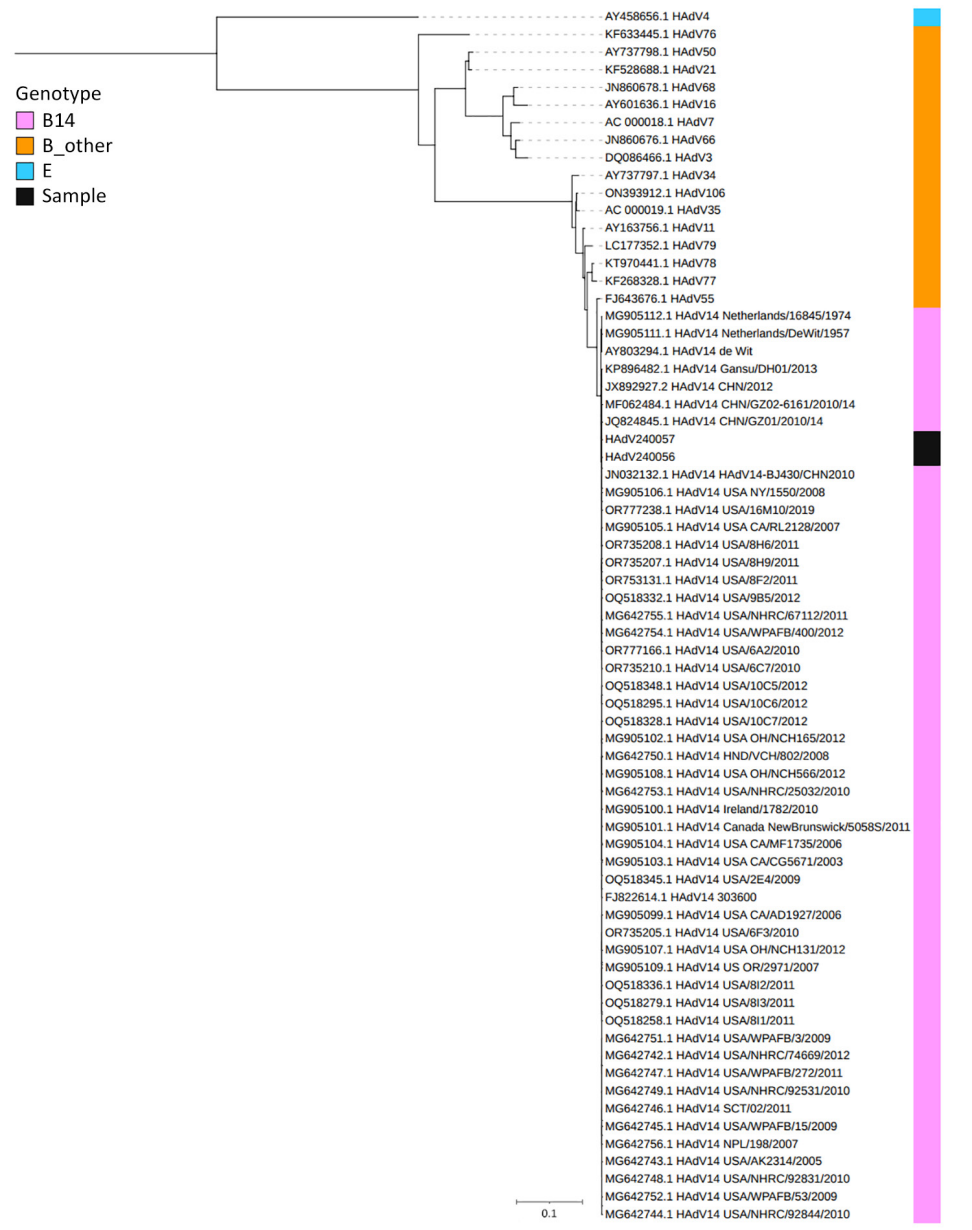
Phylogenetic analysis of HAdV complete sequences from patient isolates in study of acute encephalopathy associated with human adenovirus type 14 infection in 7-year-old girl, Japan, and reference sequences. The most recent reference strain was from the United States in 2019. The tree was created by IQ-TREE (*9*) using the maximum composite-likelihood methods with 1,000 bootstrap replicates. GenBank accession numbers are provided for reference sequences. Scale bar indicates number of substitutions per site.

The patient had influenza-like illness and acute encephalopathy and tested positive for HAdV-14. Although uncommon, HAdVs can cause central nervous system dysfunction. The most common clinical manifestations are febrile seizures and encephalitis (*1*,*10*). Few cases of HAdV-14–associated acute encephalopathy have been reported. We diagnosed our patient with acute encephalopathy associated with HAdV-14 infection on the basis of clinicoradiological correlation and viral detection in nasopharyngeal and fecal samples. The diagnosis of acute encephalopathy is usually made on the basis of neurologic manufestations and abnormal brain imaging findings, combined with HAdV detection in the cerebrospinal fluid or other sites (*10*). Although the definite mechanism of acute encephalopathy remains unknown, inflammatory cytokines produced in response to HAdV infection might induce excitotoxicity in the brain, resulting in neuronal damage (*10*).

According to the national surveillance system in Japan, only 2 cases of HAdV-14 have been reported in the past 40 years (*8*). The source of this patient’s HAdV-14 exposure is unknown. The isolate showed a high similarity to strains isolated in the United States in 2019, despite the patient having no history of contact with international travelers from countries with HAdV-14 outbreaks. Because HAdV is excreted in feces over a long period and is resistant to dessication and alcohol, community outbreaks are difficult to control (*1*). Because HAdV-14 is highly contagious and has high mortality rates, understanding its molecular evolution and clinical manifestations are key to prepare for future outbreaks. This case suggests that HAdV surveillance in Japan, including HAdV-14 surveillance, should be strengthened.
